# Fibrous Tumor of the Cervical Canal

**Published:** 1888-07

**Authors:** J. W. Bailey

**Affiliations:** Gainesville, Ga.


### FIBROUS TUMOR OF THE CERVICAL CANAL.

BY J. W. BAILEY, M. D., GAINESVILLE, GA.

This case interested me very much, as I have never seen a similar one. The rapidity of the recovery after the operation was very gratifying.

The patient gave the following history:

Mrs. W., from Tennessee—Began to suffer with these symptoms three or four months after birth of child, which occurred two years ago. Menstruation regular and normal with the exception of clots during and pains several days before its occurrence; profuse and continuous leucorrhœa; cannot lie on either side; is only comfortable on back; suffers with pains down back and thighs as far as knees; frequent severe headaches; draging pains in lower part of abdomen when standing or walking.

Upon examination found the following condition: Subinvolution, slight anteversion and the uterus very much enlarged. To my surprise I also found a growth, which at first I thought was interstitial, extending from external to internal os, attached to the anterior surface of the cervical canal, projecting from and occupying the external os.

I think the enlargement of the uterus and subinvolution due to the child-birth and not to the tumor.

After a few days preparatory treatment, I operated on the fifth day after examination.

Patient was etherized and placed in Sims' position; used Sims' speculum and drew down uterus with double tenaculum. After a tedious dissection succeeded in removing tumor entire. Hemorrhage was very slight. Washed out cervix and vagina with sol. bichlor. mer. (1-3,000) and packed cervix with cotton tampon saturated in same solution.

Tampon was removed daily and vagina washed out every other day with above solution.

Patient was confined to bed five days. There was no elevation of either pulse or temperature during treatment. In a week after operation patient was able to lie on either side with perfect comfort. Twelve days after operation, began local treatment for other troubles.

Now, after two months' treatment, uterus is normal in size; there is no subinvolution, and the leucorrhœa has entirely disappeared. Patient is able to stand and walk where she wishes without any inconvenience, and feels perfectly well in every way. Sent her home a few days ago cured.

The growth, upon close examination, proved to be fibrous; it was an inch and a quarter in length, half an inch in circumference, almost perfectly round and tapered almost to a point internally.

